# The implementation of an external quality assurance method for point- of- care tests for HIV and syphilis in Tanzania

**DOI:** 10.1186/1471-2334-13-530

**Published:** 2013-11-09

**Authors:** Pieter W Smit, David Mabey, Thomas van der Vlis, Hans Korporaal, Julius Mngara, John Changalucha, Jim Todd, Rosanna W Peeling

**Affiliations:** 1 Leiden Cytology and Pathology Laboratory, Leiden, Netherlands; 2 London School of Hygiene & Tropical Medicine, London, UK; 3 National institute for Medical Research, NIMR Mwanza, Mwanza, Tanzania

## Abstract

**Background:**

External quality assurance (EQA) programmes, which are routinely used in laboratories, have not been widely implemented for point-of- care tests (POCTs). A study was performed in ten health centres in Tanzania, to implement the use of dried blood spots (DBS) as an EQA method for HIV and syphilis (POCTs).

**Method:**

DBS samples were collected for retesting at a reference laboratory and the results compared to the POCT results obtained at the clinic. In total, 2341 DBS samples were collected from 10 rural health facilities over a period of nine months, of which 92.5% were correctly collected and spotted.

**Results:**

The EQA method was easily implemented by healthcare workers under routine conditions in Northern Tanzania. For HIV, 967 out of 972 samples (99.5%) were concordant between DBS and POCT results. For syphilis, the sensitivity of syphilis tests varied between clinics with a median of 96% (25^th^ and 75^th^ quartile; 95-98%). The specificity of syphilis POCT was consistent compared to laboratory based test using DBS, with a median of 96% (25^th^ and 75^th^ quartiles; 95-98%).

**Conclusion:**

Overall, the quality of testing varied at clinics and EQA results can be used to identify clinics where healthcare workers require remedial training, suggesting the necessity for stringent quality assurance programmes for POC testing. As Tanzania embarks on scaling up HIV and syphilis testing, DBS can be a useful and robust tool to monitor the quality of testing performed by healthcare workers and trigger corrective action to ensure accuracy of test results.

## Background

Point-of-care diagnostic tests (POCTs) are increasingly used in both developing and developed countries [1]. They allow same day testing and treatment at remote locations where no laboratory support is available. Quality control measures, which are routinely used in laboratories, have not been widely implemented for POCTs. The World Health Organization and US Centers for Disease Control and Prevention advocates the implementation of POCT with a quality assurance method in place [2].

In Tanzania, the Ministry of Health currently recommends the use of POCTs to screen pregnant women for HIV and syphilis, but an External quality assurance (EQA) method has not been implemented [3]. The use of DBS for HIV testing has been evaluated, giving comparable results to plasma [4]. Additionally, a laboratory based evaluation using Tanzanian samples showed that DBS TPPA gives comparable results to plasma [5]. Dried blood spots (DBS) have been suggested as a potential method for QA for HIV POCT [6], but this has not been implemented under routine testing services.

In this study, we implemented DBS as an EQA method for both HIV and syphilis POCT in Tanzania. The study aims were; (1) to evaluate the feasibility of using DBS as a continuous EQA method for end users in a rural setting of a developing country; (2) To review the performance of the various clinics involved in syphilis and HIV testing; (3) to compare this EQA method with proficiency panels, and (4) to make the project’s protocols available online.

## Methods

### Setting

As part of a larger study that implemented syphilis POCT in seven developing countries, the Geita District in Northern Tanzania was chosen for this study [3]. Ten out of 51 antenatal care (ANC) clinics that participated in the main study were selected based on location, type of clinic, and willingness to participate. The study was conducted in ten primary health clinics that offered ANC and HIV voluntary counselling and testing (VCT) services. To represent the various types of Tanzanian clinics providing ANC and VCT services, the district referral hospital, four health centres and five dispensaries were included in this study. The classification of clinic type is primarily based on the number of personnel and which services are offered to the patients. The study population were pregnant women and their partners. The study was conducted over a period of nine months, from January till September 2011.

### Routine services offered

The EQA method was incorporated in the national routine ANC and VCT services given at ANC clinics in Tanzania. Patients accepting HIV and syphilis testing were tested on-site using the SD Bioline syphilis 3.0 (Standard Diagnostics, Kyong gi-do, Korea) on whole blood obtained by finger-prick. The national HIV POCT testing algorithm consisted of SD Bioline HIV 3.0 (Standard Diagnostics, Kyong gi-do, Korea), Determine HIV- 1/ HIV-2 (Abbott Laboratories,Wiesbaden-Delkenheim, Germany) with Unigold (Trinity Biotech, Dublin, Ireland) as the tie-breaker test. All tests used whole blood obtained by finger prick and were performed according to the manufacturer’s recommendations. Free medical treatment was provided to all patients testing positive for syphilis. HIV positive patients were referred to Tanzanian care- and treatment centres. All services were performed according to Tanzanian guidelines.

### Implementation of EQA method in routine practice

The patients were requested to donate blood for quality control purposes and asked to provide oral consent by the health care worker (HCW). Ethical approval was obtained from ethical committees at the London School of Hygiene and Tropical Medicine and at the National Institute of Medical Research, NIMR, Tanzania. If consent was given by the patient, whole blood was obtained by finger prick according to standard protocols [2]. Whole blood was applied to the POCT and the DBS was spotted afterwards. The HCW was instructed to not perform an additional finger-prick when the DBS could not be completely filled, to minimize discomfort for the patient. Only the POCT outcome, date of testing, patient number and the clinic name were recorded on the study form.

At the start of the study, HCW that performed HIV and syphilis testing received a one- day centralised training for the collection of DBS samples. During the training, HCW learned about the method and received hands-on training of spotting blood onto filter paper, labelling DBS samples, drying, and storing DBS samples. After the training, HCW were given filter papers (Protein Savercard 903, Whatman, GE healthcare, Wisconsin, USA), desiccant bags (indicating desiccants, Brownell ltd, UK), a dry rack (GE healthcare, Wisconsin, USA) and ziplock bags (Minigrip, Netherlands) to store the DBS samples. Syphilis POCT kits (SD Bioline syphilis 3.0, Standard Diagnostics, Kyong gi-do, Korea) were purchased and transported to the hospital, from where the kits were distributed to the clinics. To minimise the workload, DBS samples were stored up to 10–15 cards per ziplock bag with desiccants at the clinic. Risk of cross contamination for serological testing was limited due to the tucked cover of the DBS cards protecting the blood spots.

To minimise the workload of the busy HCW, identification stickers were used. One sticker was placed on the DBS and the other on a registration form where the matching POCT results could be encircled. Laboratory technicians were masked to the POCT results and the registration forms containing the POCT results were entered into Epi-info v. 3.5.3 database (Centers for Disease Control and Prevention, Atlanta, USA). Interviews were conducted to obtain feedback of the HCW in June 2011. All HCW found the method easy and relatively quick to use. According to the HCW, the EQA process time including spotting, labelling and recording result varied from 2–6 minutes. As most HCW provided counselling during the waiting time before the POCT results could be read, the DBS EQA method did not cause any delay as this was done during the 15 minute waiting time.

### Shipment and laboratory methodology

The ANC registration forms and DBS samples were collected in March, June and October. Samples were counted and checked for humidity (changed desiccants if required) before shipment at ambient temperature to the laboratory. On average, the time between sampling and arrival at the laboratory where testing took place was 95 days (minimum = 62 days, maximum = 142 days). The DBS samples were transported at ambient temperature to the Leiden Cytology and Pathology Laboratory, Leiden, The Netherlands where the samples were tested. Upon arrival at the laboratory, the quality of DBS samples were reviewed and recorded according to the online protocol [7]. The DBS quality data was entered into epi-info. The DBS were placed with new desiccants in a ziplock bag and stored at 4°C until tested. To allow fast interpretation of the results, an excel file was developed that imports Epi-info data. The Excel file automatically presents data from each clinic graphically which could aid supervisors to adequately identify and provide detailed feedback to clinics requiring supervision when the EQA method is implemented. All documents are freely available online [7].

### Syphilis testing with DBS samples

*Treponema pallidum* Particle Agglutination assay (TPPA, Serodia, Tokio, Japan) was performed as described previously [5]. Briefly, 6 mm spots were punched with a DBS puncher (PerkinElmer, Greenville, SC, USA) in a 96 flat well plate. 100 μl Phosphate buffer Saline (PBS) with 0.05% tween20 was added, shaken for 2 minutes and eluted overnight at 4°C. DBS eluates were shaken for 2 min and 25 μl was transferred to a clean 96 U well plate. 25 μl specimen diluent was added, mixed thoroughly and 25 μl was transferred to a second column. 25 μl sensitized or unsensitized particles were added and agglutination was read after 2 hours incubation. Results were read independently by two technicians.

### HIV testing with DBS samples

An Enzyme Immunoassay (EIA,) Vironostika HIV EIA (Biomerieux, France), was used to review the HIV POCT results. The EIA was performed as described by the US Centers for Disease Control and Prevention (CDC) (Vironostika protocol) [8].

### Proficiency panel results

For quality assurance purposes, various measures were taken at clinic level. With every new syphilis POCT lot, a known positive and negative was tested at the clinic. Additionally, syphilis proficiency panels were made and regularly brought to clinics to assess the HCW performance. The syphilis proficiency panels consisted of four dried tubes samples (DTS) as described previously [9]. Briefly, DTS samples were made in the laboratory by drying aliquots of known positive and negative sera in small tubes and panels were sent to the clinics. The proficiency panels were sent to the clinics from July 2010 till January 2011 every month. The proficiency panels consisted of four dried tube specimens with known serostatus and changing number of positive and negative samples. At the clinic, HCWs reconstituted the DTS with PBS-tween20 buffer and the reconstituted serum was used to test with syphilis POCT. Results were recorded and sent back to the laboratory.

### Statistical analysis

Double data entry into Epi-info 3.5.3 (CDC, Atlanta, USA) was applied and data was analysed in Stata12 (Stata Corp, Texas, USA). The use of DBS for HIV or syphilis testing is not the gold standard methodology, However, for the analysis, DBS based tests were used as reference method and, to improve simplicity and readability of the manuscript, we use the terms sensitivity and specificity for syphilis and HIV POCT results, instead of positive and negative concordance between the two testing methodologies.

## Results

### DBS as EQA method

To evaluate the implementation of the EQA method across the ten antenatal care clinics (ANC), the quality of DBS samples was reviewed. In total, 2,341 samples were collected. Of these samples, 140 samples (6.07%) were excluded due to invalid syphilis POCT (60 samples) or TPPA test result (e.g. indeterminate test or test could not be performed) (64 samples), missing forms (10 samples), or the DBS sample was unusable (6 samples), either because of fungal growth or because it did not contain enough blood to obtain at least one 6 mm punch. Of the remaining 2,201 cards, containing 11,005 spots, 10,179 (92.5%) were correctly spotted. Of the 7.5% incorrect spots, 356 spots (3.2%) were empty.

### The quality of testing at Tanzanian clinics

HIV and syphilis tests are routinely performed as part of the ANC services provided to pregnant women visiting these clinics. Due to a prolonged stock-out of HIV POCT during the study period, many women (55.8%) were not screened for HIV while still receiving a syphilis test. Out of the 2201 samples included in this study, 972 (44.1%) had recorded HIV test results and 966 (99.5%) were concordant between POCT and DBS. In total, 42 patients (4.3%) were identified as positive by SD Bioline HIV POCT of which 29 were retested by Determine HIV POCT as part of the national testing algorithm. All Determine tests confirmed HIV positivity. It is unclear whether the 13 positives not confirmed by a second POCT (Determine) were identified as HIV positive or negative and they were therefore excluded from further analysis. Of the 29 positive samples, 28 (97%) were positive by DBS tests and 1 was negative. 929 (99.7%) samples were HIV POCT negative of which 3 (0.3%) were positive by DBS tests (Table 1).

**Table 1:** Syphilis and HIV POCT results compared to DBS laboratory based syphilis and HIV tests per clinic, Mwanza, Tanzania 2011

**Syphilis**				**HIV**		
**Clinic**	**POCT**	**DBS**		**POCT**	**DBS**	
		**pos**	**neg**		**pos**	**neg**
1 Hosp	pos	21	12	pos	17	1
	neg	13	376	neg	0	254
2 Disp	pos	8	7	pos	0	0
	neg	1	93	neg	0	13
3 Disp	pos	6	3	pos	2	0
	neg	1	61	neg	0	42
4 Disp	pos	12	7	pos	4	1
	neg	3	150	neg	0	67
5 Disp	pos	9	5	pos	2	0
	neg	3	96	neg	0	13
6 Disp	pos	13	6	pos	**0**	0
	neg	12	119	neg	0	24
7 HC	pos	9	4	pos	4	1
	neg	15	309	neg	1	78
8 HC	pos	14	4	pos	8	0
	neg	9	231	neg	1	248
9 HC	pos	21	5	pos	0	0
	neg	6	251	neg	0	25
10 HC	pos	17	6	pos	3	0
	neg	9	254	neg	0	162
Total	pos	130	59	pos	42	3
	neg	72	1940	neg	2	926

**Figure 1:**
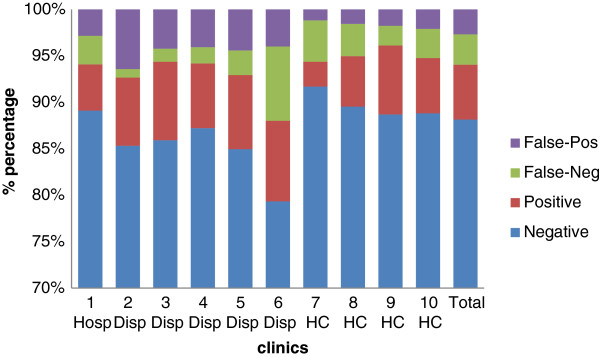
**Syphilis POCT results evaluated against DBS as reference method, Mwanza, Tanzania, 2011.** The figure provides percentages based on the total number of samples collected per clinic. Hops = Hospital, Disp = Dispensary, HC = Health centre. Pos = positive, Neg = Negative.

Out of 2,201 DBS samples collected, 189 (8.6%) were positive by syphilis POCT and 202 (9.2%) were positive by DBS TPPA. In total, 2012 samples were negative by syphilis POCT and 1999 were negative for DBS TPPA (Table 1). Using DBS TPPA as the reference method, the sensitivity of syphilis tests varied between clinics, with a median of 70% and 25^th^ and 75^th^ quartile of 61-79%. The specificity was consistent, with a median of 96% and 25^th^ and 75^th^ quartiles of 95-98% (Figure 1).

No remedial training for POCT testing or DBS collection was given during the study period. To evaluate the performance of the clinics over time, January and August, the months when the largest number of samples was collected, were compared. In January 94.5% and in August 96.7% of the samples were concordant between both methods. This finding suggests that the clinics did not perform differently at the beginning or at the end of the study.

### Proficiency panel results

To evaluate the performance of the DBS EQA method to detect underperforming clinics, we compared our results with those obtained using syphilis proficiency panels. All clinics scored 100% during November till January 2011 based on the proficiency panel (Table 2). During the 7months when proficiency panels were performed, clinic 9 scored the poorest with an average of 64% correct, while obtaining good performance in our EQA. Clinic 7 scored 79% correct in the proficiency panels while having a poor performance by our EQA (37.5%). The proficiency panel results did not correlate well with the results found by our EQA method.

**Table 2:** Samples collected per clinic and proficiency panel results per clinic

		**POCT performed per clinic**		**Proficiency panel results**		**EQA results (DBS)**			
**Clinic**	**Clinic type**	**Number of HIV tests**	**Number of syphilis tests**	Last 3 months*	**Overall (7 months)**	Sensitivity** (95% CI)		Specificity** (95% CI)	
1	District referral hospital	272	422	100%	86%	61.8%	(43.6-77.8%)	96.9%	(94.7-98.4%)
2	Dispensary	13	109	100%	89%	88.9%	(51.8-99.7%)	93.0%	(86.1-97.1%)
3	Dispensary	45	71	100%	86%	85.7%	(42.1-99.6%)	95.3%	(86.9-99%)
4	Dispensary	72	172	100%	88%	80.0%	(51.9-95.7%)	95.5%	(91–98.2%)
5	Dispensary	15	113	100%	90%	75.0%	(42.8-94.5%)	95.0%	(88.8-98.4%)
6	Dispensary	24	150	100%	93%	52.0%	(31.3-72.2%)	95.2%	(89.8-98.2%)
7	Health centre	84	337	100%	79%	37.5%	(18.8-59.4%)	98.7%	(96.8-99.7%)
8	Health centre	257	258	100%	92%	60.9%	(38.5-80.3%)	98.3%	(95.7-99.5%)
9	Health centre	25	283	100%	64%	77.8%	(57.7-91.4%)	98.0%	(95.5-99.4%)
10	Health centre	165	286	100%	95%	65.4%	(44.3-82.8%)	97.7%	(95–99.1%)
	**Total**	**972**	**2201**	**100%**	**86%**	**64.4%**	**(57.3-71%)**	**97.0%**	**(96.2-97.7%)**

*last 3 months of proficiency panel results (November - January 2011).

**sensitivity and specificity of syphilis POCT using DBS TPPA as reference.

## Discussion

In this study, DBS proved useful as an EQA method for syphilis and HIV POCT to monitor the quality of testing performed by HCW as part of routine ANC services in Tanzania. Based on our results, the quality of testing varied between the ten clinics, even though all clinics used the same syphilis POCT lots. The hospital and dispensaries seems to be performing better than the health centres when using DBS as reference method. These EQA results can be used to identify clinics where remedial training in the performance of syphilis POCTs is required.

This study showed the possibility to seamlessly integrate HIV and syphilis POCT quality assurance, making it possible to have one EQA method for preventing mother-to-child transmission of HIV services.

The DBS method has advantages over proficiency panels as this method gives insight into the quality of testing during routine patient services. Proficiency panel samples are tested by HCW with the knowledge that they are being monitored and only contain four samples. As only one proficiency panel is sent out to every clinic, only one HCW is evaluated while with DBS EQA, all HCWs are included in the EQA procedure. These reasons could potentially affect the proficiency panel result and could cause misinterpretation of the actual testing quality during routine patient services.

Compared to DBS TPPA the overall performance of the 10 clinics for syphilis POCT was lower than the manufacturer’s claim, although in agreement with an earlier evaluation in Tanzania [10]. Additionally, a laboratory based evaluation performed in Tanzania suggests that the syphilis POCT sensitivity was low compared to TPPA performed on plasma (60%) [11]. The low POCT performance found in this study should be interpreted with caution as differentiation cannot be made between discordant test result and incorrect interpretation or recording of the results by HCW. Worker turnover may be one of the reasons for low performance as the replacement worker may not have been properly trained. The strength of this study is that the EQA method can be evaluated continuously under routine clinical conditions. Because of this, the sample quality can be influenced by, for example, environmental influences during incorrect storage at the clinic or inter and national transportation of the kits.

The EQA method was easily implemented by HCW under routine conditions. Although 92.5% of the samples collected by the clinics were of good quality, humidity remained a concern. Humidity control is essential to preserve antibody and antigens stored on DBS samples and to prevent growth of mould. Storage of desiccants at the clinics for several months proved difficult as they became humid, especially during the rainy season. More suitable containers with an airtight lid should be used. Additionally, long term storage (e.g. more than 4 weeks) at clinics should be prevented as this potentially could lead to loss of samples or increase risks of unfavourable storage conditions that could lead to degradation of antibodies or antigen [12].

This study had some limitations. Because of the study design, laboratory tests were performed abroad which caused a delay in re-testing samples. It is recommended to perform re-testing at more frequent intervals and in the same country, as this will most likely be practically and financially a preferable option. Additionally, it was not feasible to monitor the costs of this EQA method.

Although the process time in the laboratory was relatively quick, this EQA method can be more costly than, for example, the use of proficiency panels. By combining this EQA for multiple infectious diseases as we did for HIV and syphilis, the economies of scope would limit the EQA costs compared to setting up two independent EQA methods.

To use this EQA method in practice, the number of samples that should be collected per clinic depends on the settings. The POCT performance (e.g. sensitivity and specificity), incidence and prevalence of the infectious disease, robustness of the test outcome, staff turnover, as well as past EQA results need to be taken into account in determining the number of samples that are required to obtain good insight in quality of POCT at the clinics [13].

## Conclusion

To the authors’ knowledge, this is the first paper describing the implementation of DBS samples as EQA method for HIV and syphilis POCT. The key findings of our work suggest the necessity for stringent quality control of POCT as the quality of testing seem to vary between clinics. On average, the sensitivity of the POCt, compared to DBS TPPA was low. The DBS EQA method potentially provides a better solution to assure the quality of POCT testing, in comparison to proficiency panels in Africa. The EQA method evaluated in this study could be easily rolled out in developing countries to improve and assure the quality of POCT testing.

## Competing interests

The authors declare that they have no competing interests.

## Authors’ contributions

PWS initiated the study, implemented the study and drafted the manuscript. TV aided in handling the samples, performed interviews and drafted the manuscript. DM and RWP provided supervision throughout the study and made major contributions to editing the manuscript. JM and JC were responsible for sample collection, sample process and revision of the manuscript before submission. JT provided guidance on the data analysis and participated in the interpretation of results. HK planned and supervised testing of the samples and collaborated in writing of the manuscript. All authors read and approved the final manuscript.

## Pre-publication history

The pre-publication history for this paper can be accessed here:

http://www.biomedcentral.com/1471-2334/13/530/prepub
